# The effectiveness of double-bar correction for pectus excavatum: A comparison between the parallel bar and cross-bar techniques

**DOI:** 10.1371/journal.pone.0238539

**Published:** 2020-09-17

**Authors:** Duk Hwan Moon, Chul Hwan Park, Mi Hyung Moon, Hyung Joo Park, Sungsoo Lee

**Affiliations:** 1 Department of Thoracic and Cardiovascular Surgery, Gangnam Severance Hospital, Yonsei University College of Medicine, Seoul, Republic of Korea; 2 Department of Radiology and Research Institute of Radiological Science, Gangnam Severance Hospital, Yonsei University College of Medicine, Seoul, Republic of Korea; 3 Department of Thoracic and Cardiovascular Surgery, Seoul St. Mary’s Hospital, College of Medicine, The Catholic University of Korea, Seoul, Republic of Korea; China Medical University Hospital, TAIWAN

## Abstract

**Purpose:**

To compare the effectiveness between parallel bar and cross-bar techniques for treating pectus excavatum.

**Methods:**

A total of 80 patients who underwent parallel bar insertion (group 1) or cross-bar insertion (group 2) were evaluated retrospectively. From the pre- and post-operative chest CT images, vertebral-level-specific pectus indices were defined as the ratio of the maximum transverse diameter to the anteroposterior diameter of the thoracic cavity at a specific vertebral level and measured at 3 levels up (3Up-PI, 2Up-PI, 1Up-PI) and 1 vertebral level down (1Down-PI) from the narrowest point. The effectiveness of double-bar correction was compared between the 2 groups using postoperative vertebral level-specific pectus index changes.

**Results:**

A total of 44 patients were enrolled in group 1, and 36 patients were enrolled in group 2. Preoperative pectus index values were not different between the 2 groups (4.5 ± 1.0 vs. 4.9 ± 1.5, *P* = 0.135). After double-bar correction, pectus index significantly decreased in both groups. There were no differences in postoperative pectus indices between the 2 groups (2.7 ± 0.4 vs. 2.6 ± 0.3, *P* = 0.197). Postoperative changes in 3Up-PI, 2Up-PI, and 1Up-PI were not significantly different between the 2 groups (*P* > 0.05). However, postoperative changes at the narrowest level and at 1Down-PI were significantly greater in group 2 than in group 1 (1.78 ± 0.85 vs. 2.32 ± 1.44, *P* = 0.009; 1.21 ± 0.70 vs. 1.70 ± 1.20, *P* = 0.009, respectively).

**Conclusions:**

Double-bar correction appears to be effective for treating pectus excavatum. The cross-bar insertion technique might be superior to the parallel bar insertion technique for correcting a wider range of deformities, especially at the lower part of the depression.

## Introduction

A great deal of progress has been made since the introduction of surgical techniques to treat pectus excavatum (PE) in 1911 by Meyer [1]. The Nuss procedure (a minimally invasive surgical technique using a metal bar) was introduced in 1998; ever since, many thoracic surgeons have adopted it to correct PE [2]. To date, there have been many reports on its benefits and effectiveness, as well as its positive long-term outcome.

Although the Nuss technique is useful, PE with chest deformity over multiple rib levels may not benefit much from single-bar insertion in one intercostal space. Generally, single-bar insertion applies a dynamic force on the vicinity of the bar, only correcting the deformity around it, and not at distal regions [3–5].

Therefore, use of additional bars should be considered, to exert force on multiple sites of the thorax, allowing the coverage of multiple rib levels. Currently, there are two known surgical techniques using a double-bar insertion approach: the parallel bar insertion technique and the cross-bar insertion technique. The choice is based on the preference of the surgeon. To the best of our knowledge, there is no study comparing the two techniques to date.

As such, the purpose of this study was to determine which of the two above-mentioned techniques may be better for treating PE. To this end, we evaluated the two techniques and compared their effectiveness.

## Materials and methods

### Study population

**Fig 1:**
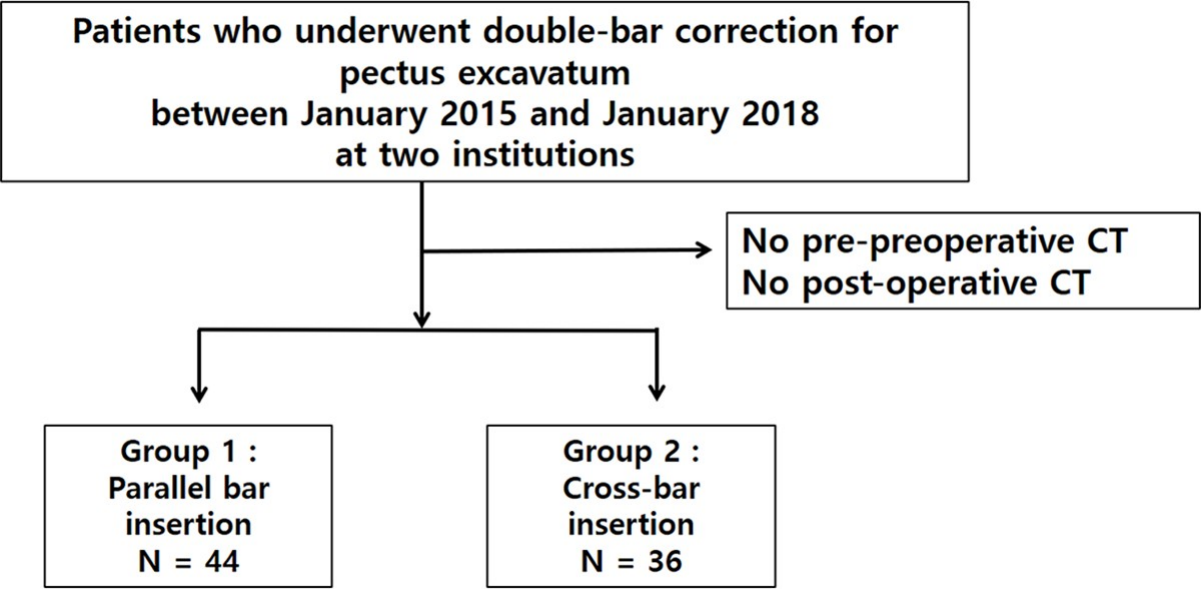
Flow chart of patient selection.

This study retrospectively evaluated patients with PE who underwent the Nuss procedure using double bars, between January 2015 and January 2018. We analyzed the following parameters: demographic characteristics, operative details, and pre- and post-operative chest computed tomography (CT) findings. PE was diagnosed by physical examination, using chest radiography and chest CT. Only patients who had undergone parallel bar and cross-bar insertions were included (Fig 1); those who had more than 3 bars placed, were excluded from this study. All bars were made of either titanium or stainless steel (Prime Medical Inc., Seoul, Korea). This study was approved by the local Institutional Review Boards of the Gangnam Severance (3-2018-0288) and the Seoul St. Mary’s Hospital (KC18REDI08). Given its retrospective design, the requirement to obtain informed consent was waived.

### Operative technique

**Fig 2:**
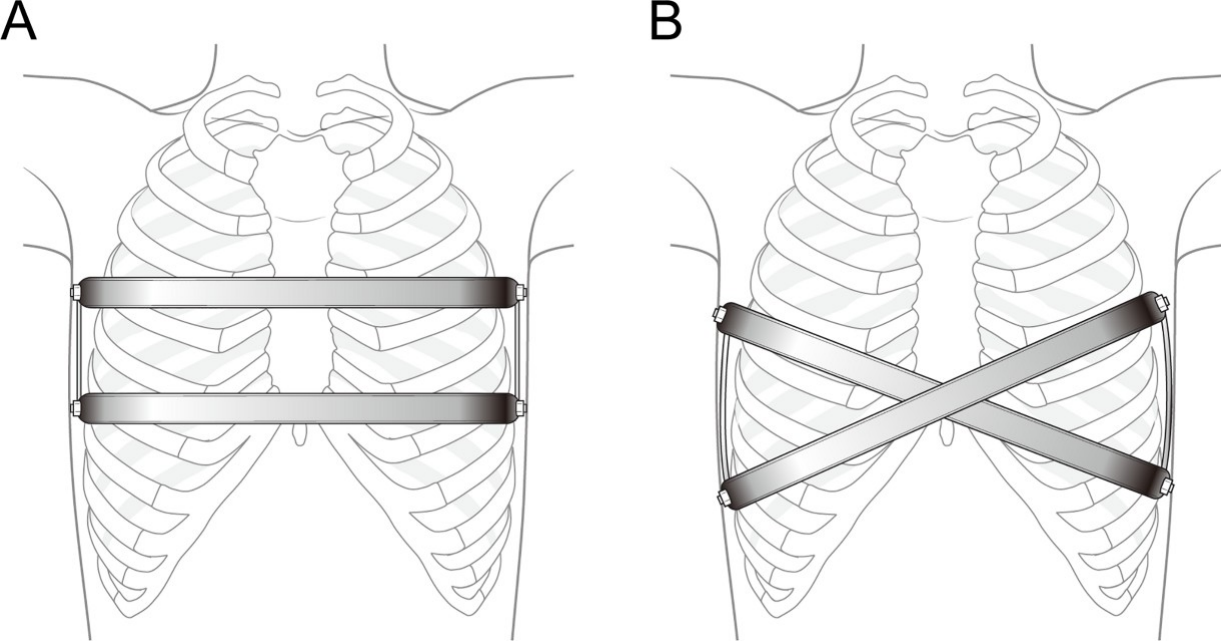
Representative images of double-bar insertion techniques. A. Parallel bar insertion, B. Cross-bar insertion.

Patients were placed in the supine position, with both arms hanging freely from the overhead crossbar on the operating table. The entire anterior chest and both groins were prepared and draped. The crane elevator was set up after draping of the thorax, and wire sutures were passed through the anterior surface of the sternum and lifted by the crane elevator. Skin incisions (1 cm in length) were performed on both lateral chest walls. The bars were configured based on the characteristics of the patient’s chest walls. In the case of cross-bar insertion, the first bar penetrated diagonally through the target area, right below the left upper intercostal spaces. The first bar was turned over and the second bar penetrated diagonally through the target area, right over the left lower intercostal spaces, using a pectus bar rotator. The point where the bars crossed lifted not only the deepest portion of the lesion, but also the entire chest wall of the patient. In the case of parallel bar insertion, the first bar was transversely inserted at the deepest point of the chest, followed by a transverse insertion of the second bar at 1 or 2 levels of intercostal spaces above the first one. Bar ends were fixed with bridge plates (Prime Medical Inc., Seoul, Korea) for both methods. We placed 2 small-caliber Hemovac drainage tubes in the pleural spaces between the wounds. Intravenous patient-controlled analgesia was routinely initiated postoperatively (Fig 2).

### CT protocol

All chest CT images were obtained with multi-detector scanners. After a scout film to determine the scan field of view, a conventional chest CT scan was performed, from the lung apex to the adrenal gland. The CT scan was performed during an inspiration in the supine position. The CT parameters were as follows: 120 kVp; 100–200 mAs; 1–3 mm slice thickness; and 1–3 mm reconstruction interval. All CT images were retrieved from a picture archiving and communication system and transferred to a commercially available reconstruction program (Aquarius iNtuition Ver. 4.4.6 TeraRecon, Foster City, CA, USA).

### CT image analysis

**Fig 3:**
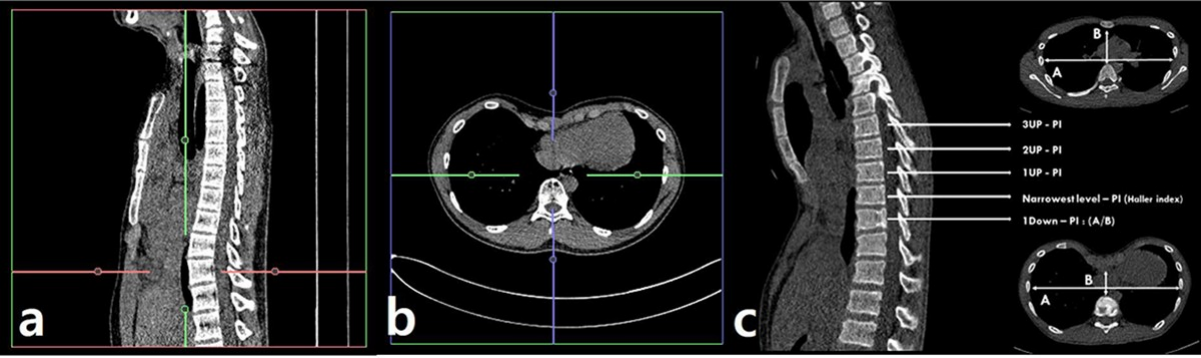
CT measurement of the vertebral-specific pectus index.

The pectus index (PI) and vertebral level-specific PIs were calculated from the pre-operative and post-operative chest CT images. The PI was defined as the ratio of the maximum transverse diameter to the shortest anteroposterior (AP) diameter of the rib cage. The vertebral level-specific PI was defined as the ratio of the maximum transverse diameter to the AP diameter of the rib cage at a specific vertebral level. For precise comparisons, vertebral level-specific PIs were measured at 3 levels up (3Up-PI, 2Up-PI, 1Up-PI) and 1 vertebral level down (1Down-PI) from the narrowest point (PI point) (Fig 3).

The effectiveness of two-bar correction was compared between the groups by using postoperative (vertebral level-specific) PI change, which was defined as follows: postoperative (vertebral level-specific) PI change = pre-operative (vertebral level specific) PI—post-operative (vertebral level specific) PI.

### Data analysis

Continuous variables were summarized as mean ± SD, and categorical variables were summarized as frequencies or percentages. Data distributions were tested using the Shapiro-Wilk test. Two-sample independent t-tests were used for the demographic differences between the 2 groups, including age, height, weight, and body mass index. The chi-square test was used for the differences in sex distribution between the two groups. Linear mixed models, with patients as the random effects and vertebral levels as the fixed effects, were used for PI and vertebral level-specific PI comparisons, considering multi-level measurements. Multivariate analyses controlling for age, sex, and body mass index as confounding factors were performed using linear mixed models. Interobserver reproducibility of PI or vertebral level-specific PI was evaluated using the intraclass correlation coefficient (ICC): ICCs of <0.40, 0.40–0.75, and 0.76–1.00 indicated poor agreement, fair to good (moderate) agreement, and excellent agreement, respectively. All statistical analyses were performed using the SPSS 20 Statistical Package for the Social Sciences (Chicago, IL, U.S.A) or SAS (version 9.4, SAS Institute Inc., Cary, NC, USA) software.

## Results

There were 44 patients in the parallel bar group (36 men and 8 women), and the median age was 16.9 ± 3.9 years. There were 36 patients (31 men and 5 women) in the cross-bar group, and the median age was 17.0 ± 4.5 years. There were no statistically significant baseline differences between the groups. Detailed demographics are summarized in Table 1.

**Table 1:** Demographic data of patients with pectus excavatum.

Variable	Parallel bar (n = 44)	Cross-bar (n = 36)	*P* value
**Age (years)**	16.9 ± 3.9	17.0 ± 4.5	0.217
**Sex (M:F)**	36:8	31:5	0.741
**Height (cm)**	169.1 ± 10.9	168.7 ± 8.9	0.965
**Weight (kg)**	54.2 ± 13.8	51.5 ± 9.7	0.333
**Body mass index (kg/m^2^)**	18.9 ± 2.6	18.0 ± 2.1	0.283

M, Male; F, Female.

**Fig 4:**
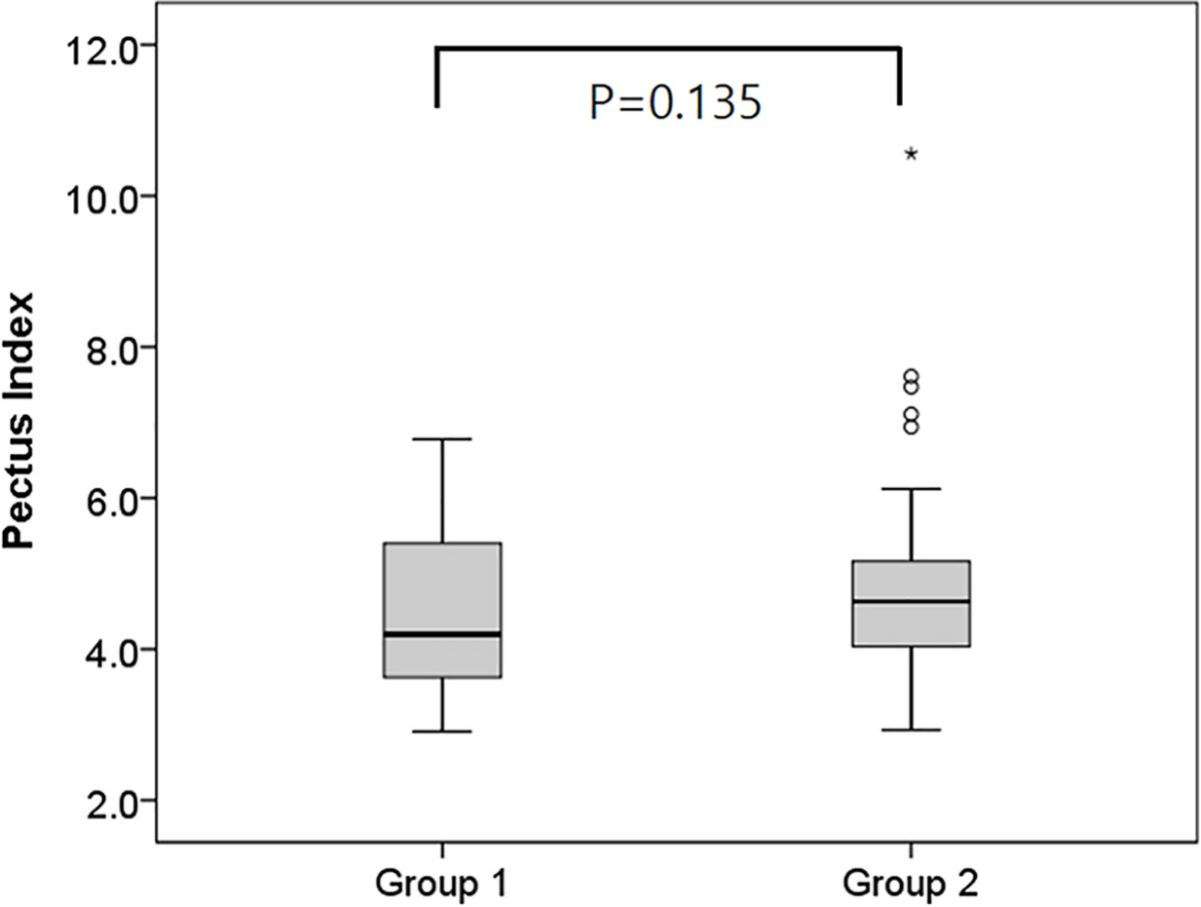
Comparison of the pre-operative pectus index assessment (Haller index) between the parallel bar and the cross-bar insertion groups.

**Fig 5:**
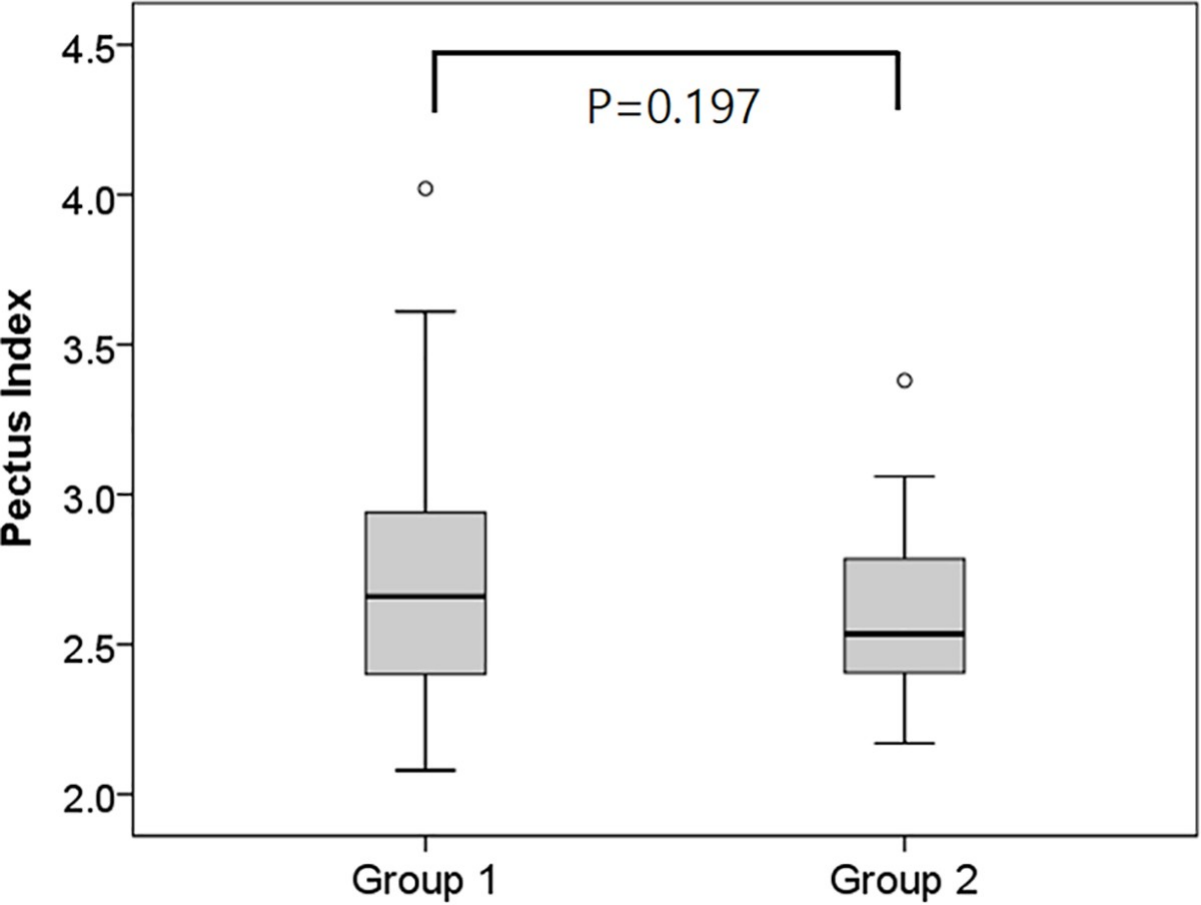
Comparison of post-operative pectus index assessment (Haller index) between the parallel bar and the cross-bar insertion groups.

There were no statistically significant differences in preoperative PI between the two groups (4.5 ± 1.0 in the parallel bar group and 4.9 ± 1.5 in the cross-bar group; *P* = 0.135) (Fig 4). After correction, postoperative PI was 2.7 ± 0.4 in the parallel bar group and 2.6 ± 0.3 in the cross-bar group, which also showed no statistically significant differences (*P* = 0.197) (Fig 5).

We compared the change in PI for both groups based on five levels. The changes in 3Up-PI, 2Up-PI, and 1Up-PI were not significantly different between the two groups (*P* = 0.217, *P* = 0.741, and *P* = 0.352, respectively). However, at the narrowest level, the cross-bar group showed a greater change in PI with statistical significance (1.78 ± 0.85 in the parallel bar group and 2.32 ± 1.44 in the cross-bar group, *P* = 0.009). Moreover, the change in 1Down-PI was also greater in the cross-bar group (1.70 ± 1.20) than in the parallel bar group (1.21 ± 0.70) (*P* = 0.009) (Table 2) [S1 File]. In the multivariate analysis, after controlling for confounding factors, changes at the narrowest level and at 1Down-PI were greater in the cross-bar group than in the parallel bar group (p < 0.05) (Table 3). The ICC for the vertebral level specific PI was 0.960.

**Table 2:** Comparison of vertebral level-specific pectus index change after double-bar correction between the parallel bar and the cross-bar insertion groups.

Variable	Parallel bar (n = 44)	Cross-bar (n = 36)	*P* value
**3Up-PI**	0.85 ± 0.62	0.61 ± 0.47	0.217
**2Up-PI**	1.09 ± 0.67	1.03 ± 0.49	0.741
**1Up-PI**	1.46 ± 0.81	1.64 ± 0.76	0.352
**Narrowest level**	1.78 ± 0.85	2.32 ± 1.44	0.009
**1Down-PI**	1.21 ± 0.70	1.70 ± 1.20	0.009

**Table 3:** Comparison of vertebral level-specific pectus index change after double-bar correction between the parallel bar and the cross-bar insertion groups using multivariate analysis.

Variable	Mean difference of vertebral level-specific PI between the parallel bar and cross-bar insertion groups	95% Confidence interval		*P* value
		Lower limit	Upper limit	
**3Up-PI**	0.210	-0.149	0.568	0.250
**2Up-PI**	0.037	-0.321	0.396	0.837
**1Up-PI**	-0.196	-0.555	0.162	0.282
**Narrowest level**	-0.531	-0.890	-0.173	0.004
**1Down-PI**	-0.546	-0.911	-0.181	0.004

## Discussion

We compared the effectiveness of parallel bar and cross-bar techniques in the treatment of pectus excavatum. The cross-bar insertion technique showed a greater change in PI at the lower part of the deformity, when compared to the parallel bar technique.

Treatment for PE has changed in terms of surgical approaches, from the Ravitch procedure, to minimally invasive techniques, such as the Nuss procedure [3, 6, 7]. The Nuss operation, introduced by Donald Nuss [8], could be used to remodel chest wall deformities without cartilage resection and has been performed throughout the world with various advantages, including a less invasive nature and better outcomes [9, 10]. However, the original Nuss procedure described a single-bar insertion, which might be insufficient for the proper correction of a heavy chest wall [7]. To overcome this issue, double-bar and multi-bar insertion techniques have been developed [4, 5, 11–13]. Although the decision to use double-bar insertion is ultimately based on the preference of each surgeon, the morphological characteristics of patients with PE may contribute to the decision. There is a lack of data on the comparison between the two techniques of double-bar insertion, the parallel bar technique and the cross-bar technique.

Yoon et al. [14] asserted that age is an important factor in determining whether double-bar technique should be used. They observed that the demand for double-bar insertion was higher among individuals in late adolescence and adults than in other age groups. Moreover, they also showed that there was greater stabilization of the bar using the double-bar technique. It is acknowledged that the single-bar technique is not ideal in adults when compared to children, especially due to the greater size and stiffness of the chest wall in adults [13].

Most patients with asymmetric PE had right-sided asymmetry, and the cross-bar technique was performed more frequently in these patients [5]. This may be because severe right-sided depression is more frequently accompanied by long-segment depression rather than simple depression.

In this study, we found that the cross-bar technique was more advantageous from a multi-level correction perspective, especially in patients with long-segment depression at the narrowest point level and one vertebral level down from the narrowest point. Depression in the lower part of the xiphoid process can be corrected using the cross-bar technique. Moreover, the crossing mechanism of the bars on both sides acts as a lever, pulling and lifting each other, allowing for a more even shape [4]. Although, in this study, no statistical significance was observed, slight depression may be present if the parallel bar technique is used on a long segment, encompassing more than two intercostal spaces. In addition, the cross-bar technique allows the surgeon to choose the range that will be lifted and prevent rib flare due to the diagonal insertion of the bars.

With respect to skin incision, the double-bar technique requires two more incisions than that in the single-bar technique. However, given that the former results in higher patient satisfaction regarding shape, the issue of additional incision scars may be negligible. In addition, the double-bar technique provides better pressure distribution, resulting in improved bar security. Moreover, the double-bar technique, when compared with the single-bar technique, may facilitate greater development of the stabilizer [13, 15].

The Haller index (HI) is universally used as an indicator for surgical treatment of PE [1, 16]. The cut-off value of HI has traditionally been set at 3.25; a value higher than the cut-off indicates surgical treatment [16, 17]. Since its introduction, many other indices have been developed [16, 18, 19]. However, the roles of these indices have been shown to be limited in determining whether surgical treatment is required. Additionally, the HI is also insufficient for determining the degree of postoperative chest wall correction. Although with some limitations, the measurement tool presented in this study may be easily applicable for evaluating the degree of postoperative chest wall correction.

Several limitations of this study should be taken into consideration. Firstly, this was not a prospective, randomized trial; thus, we were unable to compare patients with similar chest shapes preoperatively. In future studies, we aim to develop a scale measuring the degree of pectus excavatum (e.g., severity regarding deepest level, range of depressed defect, etc.) to allow for more objective comparisons. Secondly, the pain scores between the two techniques, influence of respiration, or any other physiological characteristics were not evaluated. These factors will be considered in a future study. Nevertheless, to the best of our knowledge, this is the first study to report the differences between these two techniques.

In conclusion, the double-bar insertion technique appears to be an effective modality for treating PE. In particular, the cross-bar insertion technique might be superior when compared with the parallel bar insertion technique for correcting a wider range of deformities, especially at the lower part of the depression. If patients are screened and the right candidates are chosen for treatment, this may be a good surgical option for treating PE.

## Supporting information

S1 File Attached file contains data of vertebral level-specific pectus index change after double-bar correction between the parallel bar insertion group (group 1) and the cross-bar insertion group (group 2). (XLSX)
